# Optical Clearing and Light Sheet Microscopy Imaging of Amphioxus

**DOI:** 10.3389/fcell.2021.702986

**Published:** 2021-07-26

**Authors:** Simona Machacova, Helena Chmelova, Anna Vavrova, Zbynek Kozmik, Iryna Kozmikova

**Affiliations:** ^1^Laboratory of Transcriptional Regulation, Institute of Molecular Genetics of the Czech Academy of Sciences, Prague, Czechia; ^2^Light Microscopy Core Facility, Institute of Molecular Genetics of the Czech Academy of Sciences, Prague, Czechia

**Keywords:** amphioxus, light sheet microscopy, clearing technique, whole mount immunohistochemistry, photoreceptor, acetylated tubulin, melanopsin

## Abstract

Cephalochordates (amphioxi or lancelets) are representatives of the most basally divergent group of the chordate phylum. Studies of amphioxus development and anatomy hence provide a key insight into vertebrate evolution. More widespread use of amphioxus in the evo–devo field would be greatly facilitated by expanding the methodological toolbox available in this model system. For example, evo–devo research on amphioxus requires deep understanding of animal anatomy. Although conventional confocal microscopy can visualize transparent amphioxus embryos and early larvae, the imaging of later developmental stages is problematic because of the size and opaqueness of the animal. Here, we show that light sheet microscopy combined with tissue clearing methods enables exploration of large amphioxus specimens while keeping the surface and the internal structures intact. We took advantage of the phenomenon of autofluorescence of amphioxus larva to highlight anatomical details. In order to investigate molecular markers at the single-cell level, we performed antibody-based immunodetection of melanopsin and acetylated-α-tubulin to label rhabdomeric photoreceptors and the neuronal scaffold. Our approach that combines light sheet microscopy with the clearing protocol, autofluorescence properties of amphioxus, and antibody immunodetection allows visualizing anatomical structures and even individual cells in the 3D space of the entire animal body.

## Introduction

Cephalochordata (also called lancelets or amphioxi) represent the earliest chordate lineage (Delsuc et al., 2006) and share with vertebrates a similar body plan. Therefore, amphioxus is the best animal model to elucidate the origin and evolution of chordates. The studies that investigate the early development of amphioxus embryos prevail over the ones examining the ontogenesis from late larvae to adult animals. Many techniques for imaging early embryos give poor results when applied to late larvae. And yet, asymmetric amphioxus larvae possess plenty of remarkable characteristics such as a preoral pit, an extended oral opening located on the left side, a club-shaped gland, an endostyle, an unpaired gill slits on the right side (while posterior gill slits rotate to medioventral position in early larvae), a Hatschek’s groove, a Hatschek’s nephridium, a Räder organ (a wheel organ), and an anus moving from the originally right position to medioventral position and then to the left (Hatschek, 1881; Willey, 1891, 1894; Conklin, 1932; Stokes and Holland, 1995). The specimens at later developmental stages exhibit high-density tissue and lose optical transparency typical of amphioxus embryonic and early larval stages. One-month-old larva is already partially opaque, and such techniques as whole-mount *in situ* hybridization or immunohistochemistry followed by confocal microscopy imaging become challenging. In the past, large-specimen analysis and visualization was mainly performed by using mechanical sectioning and imaging of individual slices. Nowadays, single plane illumination microscopy (SPIM; or light sheet microscopy) combined with tissue clearing allows significantly faster examination of a large specimen while retaining the key context of the surrounding tissue (Huisken and Stainier, 2009; Masson et al., 2015; Nie et al., 2020). The desired transparency is achieved by elimination of light scattering on the interface between environments with various refractive indexes, mostly lipid deposits and light-absorbing substances. CUBIC is a hydrophilic clearing method that enables effective tissue clearing in two steps (Susaki et al., 2015). First, CUBIC1 reagent washes out lipids, and second, CUBIC2 substitutes the space instead of missing lipids to equilibrate the refractive index of the tissue. The advantages of the method are safety, usage of commonly available chemicals, and simplicity. In addition, the CUBIC method allows combining tissue clearing with immunohistochemistry and endogenous fluorescence detection.

In this study, we applied the CUBIC clearing method followed by light sheet microscopy imaging to examine 1–, 3–, and 6-month-old *Branchiostoma floridae* specimens. It has been reported that amphioxus possesses its own green fluorescent proteins (GFPs) ubiquitously distributed in larvae. In metamorphic juvenile, GFPs are located in the anterior body portion, predominantly in the support cells of oral cirri, more diffusely in the epidermis (mainly in the anterior and posterior ends of the animal) (Deheyn et al., 2007; Bomati et al., 2009). We analyzed the autofluorescence of larvae and juvenile specimens exposed to four excitation wavelengths (405, 488, 561, and 638 nm). In addition, we performed immunohistochemistry (IHC) staining for acetylated tubulin to visualize the neural net and for melanopsin to label rhabdomeric photoreceptor cells (Koyanagi et al., 2005; Pergner and Kozmik, 2017).

## Materials and Equipment

### Animal Culture, Spawning Induction, and Fixation

#### Equipment

• Room with controlled temperature and light/dark cycle
• Sea water (Bremerhaven, Alfred-Wegener-Institut)
• Saltwater tank with mechanical filtration and UV sterilizer (AQUA SCHWARZ)
• 5 L tank
• Algae (*Isochrysis lutea*, *Phaeodactylum tricornutum*)
• Plastic cups
• Red-light flashlight
• Stereomicroscope
• 5 and 15 cm plastic Petri dishes coated with 1% agarose to prevent sticking the eggs to the bottom
• Manual centrifuge (∼3,000 rpm)
• 15 ml centrifuge tubes
• Rotator-nutator shaker

#### Stock solutions

• 16% paraformaldehyde aqueous solution, EM grade, 10 ml ampoule
• MOPS solution [0.1 M 3-(N-morpholino)propanesulfonic acid, 2 mM MgSO_4_, 1 mM EGTA, 0.5 M NaCl], pH 7.5, storage at –20 °C

#### Working solution

• Filtered sea water mixed with distilled water (7:1)
• 4% MOPS–PFA, pH 7.5
• 100% methanol

### Whole-Mount Immunohistochemistry, Clearing, and Mounting

#### Equipment

• 10 ml Erlenmeyer flask (for clearing the agarose column)
• Modeling clay
• Glass capillary for light sheet microscopy (size 2, 3)

#### Stock solutions

• 100% methanol
• 10× TBS buffer (Tris base 200 mM, NaCl 1.5 mM, pH 7.6)
• 10% Triton X-100
• 10% bovine serum albumin (BSA)
• 100% normal donkey serum (NDS)
• Mouse anti-melanopsin primary antibody, generated in our laboratory (Bozzo et al., 2017)
• Mouse anti-melanopsin primary antibody, generated by Koyanagi et al. (2005)
• Rabbit anti-acetyl-α-tubulin antibody (Cell Signaling #5335, Danvers, MA, United States)
• Donkey anti-mouse Alexa 647 (Thermo Fisher A-31571, Waltham, MA, United States)
• Donkey anti-rabbit Alexa 488 (Life Technologies A-21206, Carlsbad, CA, United States)
• 4′,6-Diamidine-2′-phenylindole dihydrochloride (DAPI)
• Urea
• N,N,N′,N′-Tetrakis(2-hydroxypropyl)ethylenediamine (4NTEA)
• Triethanolamine (TEA)
• Sucrose
• Triton X-100

#### Working solutions

• Methanol series [70, 50, 25% (wt/wt)]
• 1× TBT buffer (1 × TBS, 0.1% Triton X-100), filtered
• Blocking solution [4% (wt/wt) BSA, 40% (wt/wt) NDS in 1× TBT]
• 1.7% low gelling temperature agarose dissolved in distilled water
• CUBIC1—clearing solution [35% (wt/wt) dH_2_O, 25% (wt/wt) urea, 25% (wt/wt) 4NTEA, 15% (wt/wt) Triton X-100]; add in order and allow each reagent to dissolve; stir over low heat (∼42 °C)
• CUBIC2—refractive index matching solution [15% (wt/wt) dH_2_O, 25% (wt/wt) urea, 10% (wt/wt) TEA, 50% (wt/wt) sucrose, 0,1% (wt/wt) Triton X-100]; add in order and allow each reagent to dissolve; stir over low heat; final refractive index 1.473; store for 1–2 months at RT; strong smell of ammonia indicates expired solution

### Light Sheet Microscopy Imaging

• Zeiss Lightsheet Z.1 microscope (Carl Zeiss AG, Oberkochen, Germany)
• 10×/0.2 illumination objective
• Detection objective Clr Plan-Neofluar 20×/1.0 Corr nd = 1.45 suited for CUBIC2 clearing media
• Camera PCO.edge 5.5 (sCMOS), 6.5 μm pixel
• Storage for data imaging (∼160 TB)
• Stereomicroscope for sample preparation

#### Software requirements

• ZEN 3.1 (Carl Zeiss AG, black edition)
• Imaris Stitcher ×64 9.6.0 software (Bitplane, Zurich, Switzerland)
• Imaris ×64 9.6.0 software (Bitplane)
• Adobe Illustrator CS6
• Fiji ImageJ

### Methods

#### Animal Culture and Fixation

Adults of *B. floridae* were collected in Old Tampa Bay, Florida, during the spawning season and were cultured at the Institute of Molecular Genetics of the Czech Academy of Sciences (Prague, Czechia). The maintenance of amphioxus culture and spawning induction was described previously (Pergner et al., 2020). Embryos and larvae were raised at 25 °C. Initiation of feeding started 2 days post fertilization with *Isochrysis lutea* and *Phaeodactylum tricornutum*. The animals were fed daily. The larvae were kept in a 90-mm tissue culture dish for 1 month in the dark without day/night cycle. After 1 month, the animals were kept in a 14/10-h day/night cycle. Two-month-old juveniles were transferred to boxes with a volume of 1.5 L and fed daily with *Isochrysis lutea.* Larvae and juveniles in desired stages of development were fixed with 4% MOPS–PFA for 15 min on ice and transferred into 100% methanol, followed by five washes in 100% methanol, 20 min each. Samples were stored at –20 °C.

#### Tissue Clearing and Immunohistochemistry Staining

(1) Place samples to a four-well plate and transfer them from methanol to TBT through a series of washings with TBT containing 70, 50, and 25% of methanol (for 15 min each, volume 0.5 ml, agitating).
(2) Wash three times in TBT for 15 min (in 0.5 ml, agitating).
(3) With continued agitation, add a few drops of CUBIC1 solution every 30 min. When the well is full, replace the liquid with fresh CUBIC1. Leave the specimens in CUBIC1 on the rotating shaker at room temperature overnight or longer until the total tissue is cleared (depending on the specimen size, it may last for 12–48 h). Replace CUBIC1 by a fresh one every 24 h.

*Attention*: Fast direct transfer of larger samples into CUBIC1 causes irreversible bending of individuals.

(4) Remove half of the liquid and add the same volume of TBT, then wash the cleared samples for 30 min with agitation.
(5) Wash the samples in TBT alone five times for 20 min with agitation.

(*Optional IHC steps follows*)

(6) Block the specimens dedicated to IHC staining in blocking solution for at least 1 h at room temperature with agitation.
(7) Incubate the samples in TBT with 10% BSA containing primary antibodies overnight at 4 °C with agitation.

In this study, anti-acetyl-α-tubulin (1:500) and anti-melanopsin (1:250) were used. Mouse polyclonal antibody was generated against amphioxus melanopsin as described by Bozzo et al. (2017) using a peptide derived from the C-terminus of *B. floridae* protein.

(8) The next day, wash the samples five times in TBT for 20 min with agitation.
(9) Incubate samples in TBT containing 10% BSA with secondary antibodies for 3 h at room temperature with agitation.

In this study, donkey anti-mouse Alexa 647 (1:500) and donkey anti-rabbit Alexa 488 (1:500) antibodies were used.

(10) Wash the samples in TBT five times for 20 min with agitation.

(*End of IHC steps*)

(11) Stain nuclei with DAPI (1:1,000) in TBT overnight at 4 °C with agitation.
(12) Wash the samples in TBT three times for 20 min at room temperature with agitation.
(13) Transfer the specimens into CUBIC2 solution in the same way as in the case of CUBIC1.
(14) Samples can be stored in CUBIC2 at room temperature until imaging.

*Attention*: Storage longer than 1 month causes crystallization of CUBIC2, which can be turned back to the liquid state by adding TBT and gentle mixing.

(15) For imaging, immerse the samples into warm agarose (about 42 °C) and suck them into a glass capillary. Choose the size of the glass capillary corresponding to the size of the specimen.
(16) To adjust the agarose refractive index, it has to be incubated in CUBIC2 as well. After agarose polymerization, transfer the capillary into a 10-ml Erlenmeyer flask containing CUBIC2 solution and attach the capillary to the neck of the flask by modeling clay. Gently push out the agarose column containing specimens into CUBIC2 solution. Left the samples in a dark room for at least 24 h.

#### Microscopy Imaging and Data Processing

Samples were imaged under a Zeiss Lightsheet Z.1 microscope (Carl Zeiss AG) using the software ZEN 3.1 (black edition, Carl Zeiss AG) and equipped with two 10×/0.2 illumination objectives (for dual-sided illumination), detection objective Clr Plan-Neofluar 20×/1.0 Corr nd = 1.45 suited for CUBIC2 clearing media and two cameras pco.edge 5.5 (sCMOS). Online dual side fusion in ZEN 3.1 (black edition) was used to fuse left and right illuminated images. Individual channels were imaged sequentially in a switch mode between z-stacks (i.e., first, the z-stack image of one fluorophore channel is completed, then it switches to the other). The excitation and emission wavelengths were used in individual channels as follows: blue (excitation 405 nm, emission 420–470 nm), green (excitation 488 nm, emission 505–545 nm), red (excitation 561 nm, emission 575–615 nm), and magenta (excitation 638 nm, emission LP 660 nm).

The specimens were imaged according to the conditions described in Supplementary Table 1. The obtained tiles (squares in a regular grid subdividing the image) were stitched in Imaris Stitcher ×64 9.6.0 software (Bitplane). Visualization of the final images was done in Imaris ×64 9.6.0 software version (Bitplane) and Fiji ImageJ software. Postprocessing of the data was done on specialized analysis computers (Supplementary Table 2). Final panel figures were assembled in Adobe Illustrator CS6.

## Results

### Tissue Clearing of Amphioxus

**FIGURE 1:**
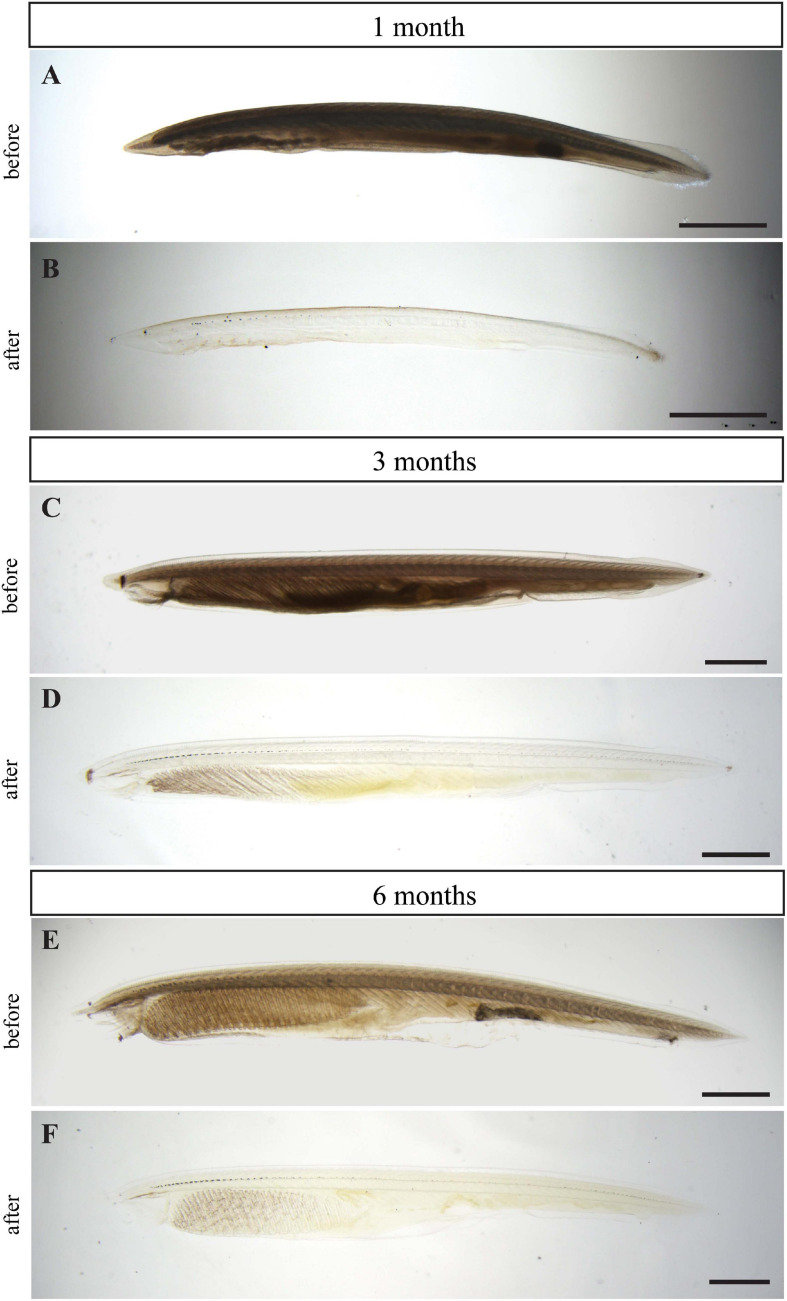
Clearing effect of CUBIC technique on larvae and juveniles of *B. floridae*. One-month-old larva before **(A)** and after clearing **(B)**, 3-month-old juvenile before **(C)** and after clearing **(D)**, and 6-month-old juvenile before **(E)** and after clearing **(F)**. Larvae and juveniles depicted before and after clearing came from the same batch and were raised in the same conditions. Wide field microscopy with transmitted light was used for imaging all specimens. Scale bar 0.5 mm.

The embryos of *B. floridae* are transparent during the first weeks of development. As the larva grows, it loses its transparency and examination of the whole-mount morphology becomes challenging. To overcome the low transparency of older samples, we applied the hydrophilic tissue clearing method CUBIC. Five individuals of 1-month-old larva along with 3– and 6-month-old juveniles were selected for the following experiments: solely CUBIC clearing or combined with IHC. The intact and cleared larvae and juveniles were observed under a conventional light stereomicroscope (Figure 1). The clearing procedure remarkably increased the transparency of the animal specimens. The pigmented cells forming the dorsal ocelli or frontal eye became distinctly visualized in the background of cleared animals.

### Autofluorescence and Acetylated Tubulin Staining of 1-Month-Old Larvae

**FIGURE 2:**
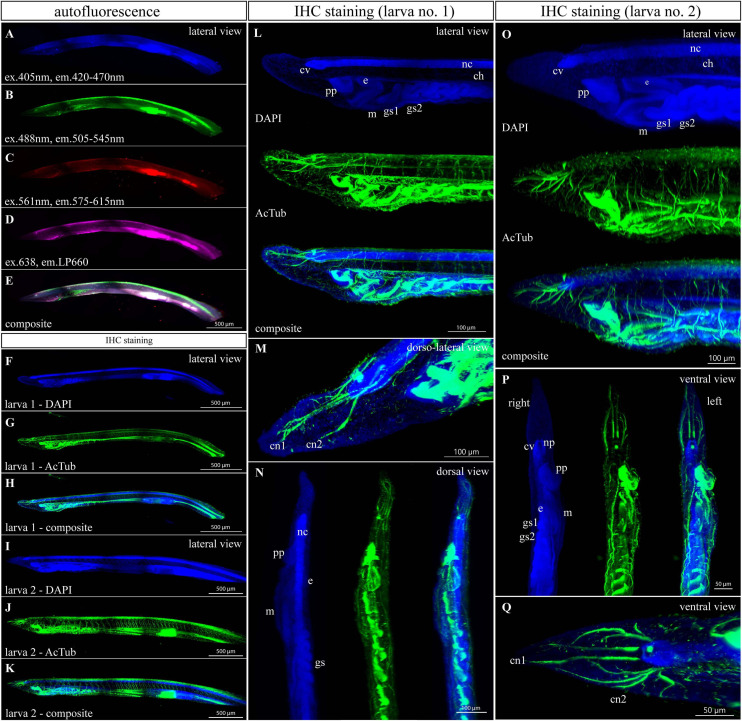
Autofluorescence and anti-acetylated-α-tubulin staining of a 1-month-old amphioxus larva. **(A–D)** Individual scan of larva autofluorescence emitted in blue **(A)**, green **(B)**, red **(C)**, and magenta channels **(D)**. **(E)** Composite image of previous scans. The larva scanned for autofluorescence was about 2.9 mm long and had seven unpaired gill slits. **(F–Q)** Immunofluorescent staining of acetylated tubulin in 1-month-old larvae. **(F–K)** Whole-mount images of two different individuals. Larva 1 was about 2.7 mm long and had seven unpaired gill slits. Larva 2 was about 3.5 mm long and had eight unpaired gill slits. **(L)** Detail of the anterior portion of larva 1 from the left side view. **(M)** Detail of cranial nerves of larva 1 from the dorsolateral view. **(N)** Detail of the anterior portion of larva 1 from the dorsal view. **(O)** Detail of the anterior portion of larva 2 from the left view. **(P)** Detail of the anterior portion of larva 2 from the ventral view. **(Q)** Detail of cranial nerves and a cerebral vesicle of larva 2 from the ventral view. Larvae 1 and 2 came from the same batch and were raised in the same conditions. All fluorescence images represent 3D reconstruction of the whole body mass. The regular areas of low signal intensity are caused by lower intensity of light falling on the camera chip margins during the tile imaging. ch., notochord; c.n.1/2, first and second cranial nerves; c.v., cerebral vesicle; e., endostyle; g.s.1/2, first and second gill slit; m., mouth; n.c., neural cord; np., neuroporus; p.p., preoral pit. Individual channels were imaged sequentially in the track switch mode—z-stack.

Endogenous fluorescence constitutes both an advantage and an obstacle for imaging procedures. On the one hand, autofluorescence may serve as an endogenous marker of certain structures or cells. On the other hand, endogenous fluorescence could interfere with the desired staining in a given stage of development. Therefore, we explored the amphioxus endogenous fluorescent signal of cleared larvae and juveniles. One-month-old larva with seven unpaired gill slits exhibited a low level of autofluorescence excited by 405, 488, 561, and 638 nm laser wavelengths. In all spectral channels, the fluorescent intensity was the strongest in the gut (Figures 2A–E) and frontal eye (Figures 2B,D,E). Blue autofluorescence was detected almost homogeneously throughout the tissue (Figure 2A). Emission induced by 488 and 638 nm laser was predominantly present in the most dorsal tissue (fin rays lying in the fin chambers), gut, and branchial apparatus (Figures 2B,D). Red autofluorescence was the strongest in the gut (Figure 2C). Next, we performed combined IHC staining of acetyl-α-tubulin with DAPI (Figures 2F–Q and Supplementary Videos 1, 2). The laser power required to excite DAPI or Alexa 488 was three times lower than in the case of endogenous fluorescence. Thus, the autofluorescence could not hinder the imaging of exogenous markers. Probably due to the higher density of nuclei, DAPI staining showed stronger intensity in the neural cord with brain vesicle, neuropore oral opening and branchial apparatus, endostyle, and gut, including the ilio-colon ring (Figures 2F,H,I,K). Staining with anti-acetyl-α-tubulin antibody clearly visualized the preoral pit, endostyle, gill slits, frontal eye, and nervous system throughout the whole specimen (Figures 2G,H,J–Q and Supplementary Videos 1, 2).

The regular dark regions in Figures 2A–K represent a processing artifact caused by stitching the tiles into one image because of lower light intensity in the camera chip margins. This effect could be avoided by using only the central part of the chip. In this case, the number of tiles would have to be doubled and the imaging time of the whole sample would be increased. The need of stitching can be avoided by usage of real-time adjustable tiling light sheet selective plane illumination microscopy (TLS-SPIM) or multiangle-resolved subvoxel selective plane illumination microscope (Mars-SPIM) (Fu et al., 2016; Nie et al., 2020).

### Autofluorescence and Acetylated Tubulin Staining of a 3-Month-Old Juvenile

**FIGURE 3:**
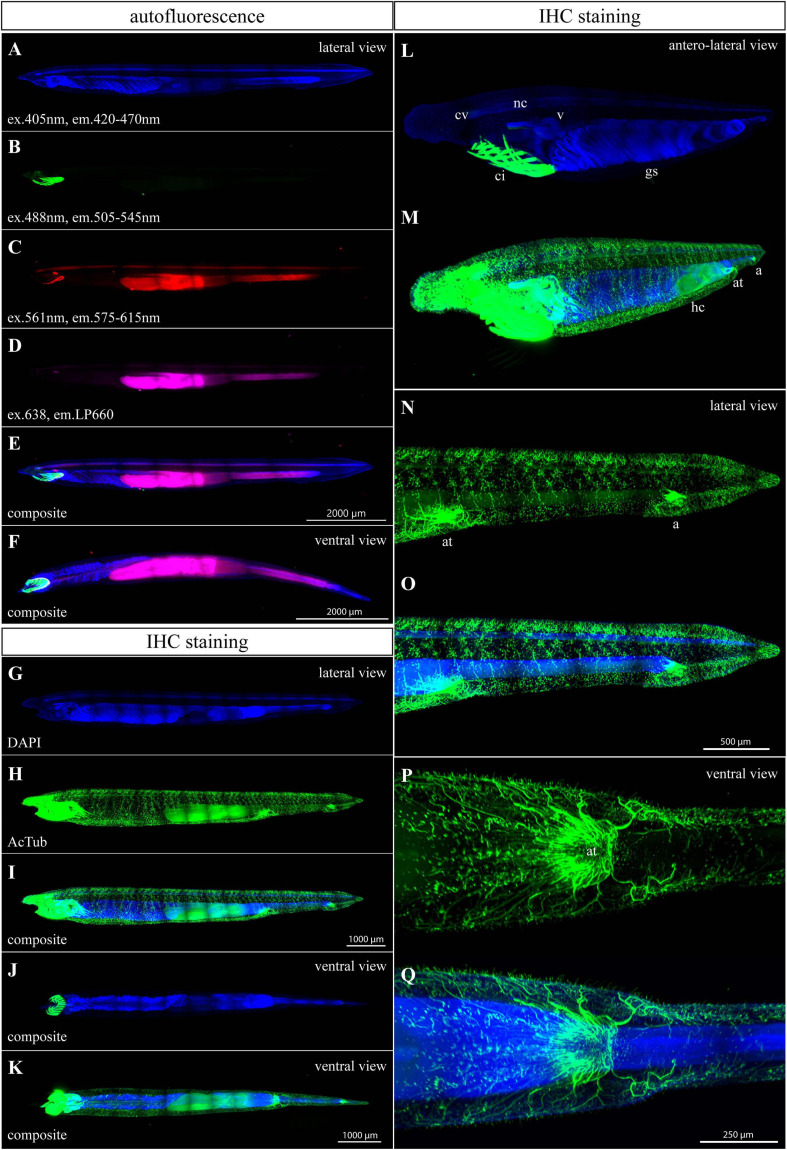
Autofluorescence and anti-acetylated-α-tubulin staining of a 3-month-old amphioxus juvenile. **(A)** DAPI staining of a specimen examined for autofluorescence. **(B–F)** Individual scans of juvenile autofluorescence emitted in green **(B)**, red **(C)**, and magenta channels **(D)**. **(E,F)** Composite image of previous scans from the lateral **(E)** and ventral view **(F)**. The specimen scanned for autofluorescence was about 8 mm long. **(G)** DAPI staining of the specimen in **(H–I)**. **(H–N)** IHC staining of acetylated tubulin. The stained juvenile was 8 mm long. **(J,K)** Ventral view of immunostained juvenile. **(L,M)** Anterolateral view of the anterior portion of immunostained juvenile. Differential adjustment of brightness/contrast of the green channel was required to demonstrate the intensity of strong endogenous GFP signal in cirri **(L)** compared with the staining of acetylated tubulin **(M)**. **(N,O)** Detail of the posterior portion of immunostained juvenile, from the lateral view. **(P,Q)** Ventral view of atriopore innervation. All fluorescence images represent 3D reconstruction of the whole body mass. The regular areas of low signal intensity in the images are caused by lower intensity of the light falling on the camera chip margins during the imaging of the tiles. a., anus; at., atriopore; ci., buccal cirri; c.v., cerebral vesicle; g.s., gill slits; h.c., hepatic cecum; n.c., neural cord; v., velum.

Furthermore, we tested a 3-month-old juvenile after metamorphosis with 28 pairs of gill slits and roughly 8 mm in length (Figure 3). Three-month-old juveniles exhibit right-side position of hepatic diverticulum and medioventral position of both atriopore and anal opening. In general, the detected autofluorescence had similarly low intensity as in the case of 1-month-old larvae except for the appearance of signal in the oral apparatus (compare Figures 2A–E, 3B–F). Autofluorescence emitted in the blue spectrum was almost undetectable. Therefore, DAPI staining was performed to get the outline of the whole-mount specimen (Figure 3A). The DAPI signal was found in all tissues and the strongest intensity was detected in the neural tube, velum, gill slits, and gut. Endogenous GFPs of amphioxus were strongly expressed in oral cirri (Figures 3B,E,F). A lower green signal was also detected in the frontal eye. The frontal eye, basis of the oral apparatus, neural tube, hepatic diverticulum, and gut were noticeable in the red spectrum (Figure 3C). The autofluorescence signal in the far red spectrum was detected in the frontal eye, hepatic diverticulum, and gut (Figures 3D–F). Immunohistochemistry staining for acetyl-α-tubulin revealed advanced progressive branching of neuronal axons when compared with younger larvae (compare Figures 2F–Q with Figures 3G–Q and Supplementary Video 3). Acetylated tubulin marked the neural net with axons extending from the neural tube in dorsal and lateroventral directions. Strong innervation was present in the rostrum, frontal eye, velum, hepatic diverticulum, nerve net surrounding the atriopore, and anal opening (Figures 3H–Q and Supplementary Video 3). The strong endogenous GFP signal in the cirri interfered with Alexa 488 from IHC staining (Figures 3J–M and Supplementary Video 3). Therefore, when researchers are interested in studying the oral apparatus in juvenile or adult amphioxus, they should use a secondary antibody with a red or far red fluorophore.

### Autofluorescence and Acetylated Tubulin Staining of a 6-Month-Old Juvenile

**FIGURE 4:**
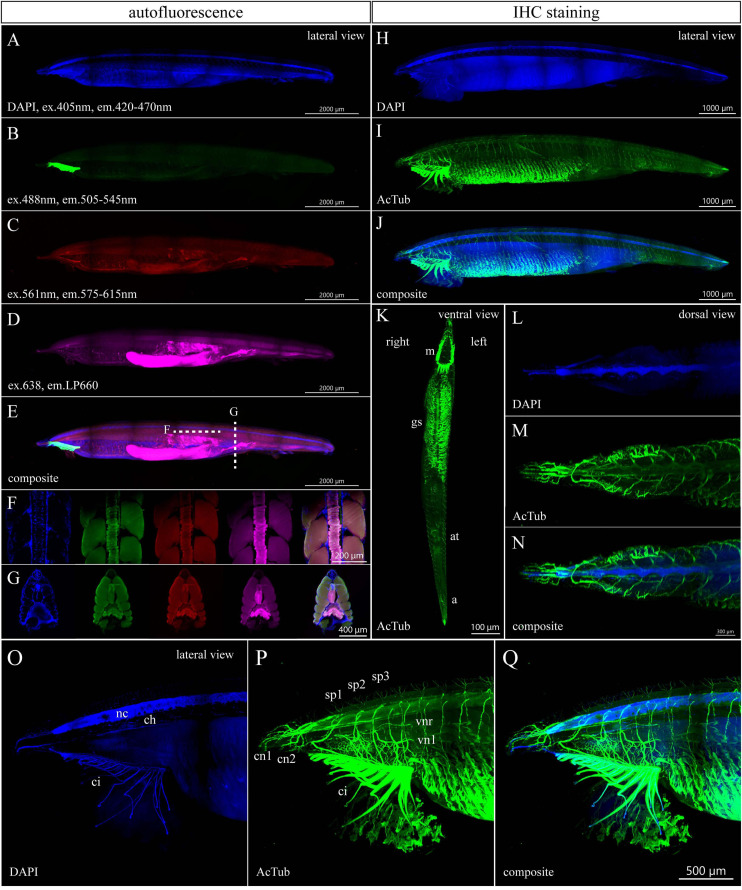
Autofluorescence and anti-acetylated-α-tubulin staining of a 6-month-old amphioxus juvenile. **(A)** DAPI staining of a 6-month-old juvenile with a length of 10.5 mm. **(B–E)** Autofluorescence of the same individual emitted in green **(B)**, red **(C)**, and magenta channels **(D)**. **(E)** Composite image from the lateral view. **(F,G)** Subset of horizontal **(F)** or transversal **(G)** optical slices of the specimen from **(A–E)** in the area with notochord showing the DAPI staining and autofluorescence in green, red, and magenta channels with a composite of all above images (from left to right). **(H–Q)** IHC staining of acetylated tubulin of a 6-month-old juvenile with a length of 9.7 mm shown as whole mount **(H–K)** or individual portion of the body **(L–Q)**. Detail of the anterior portion of juvenile body after removal of optical slices containing buccal cirri, **(O–Q)** detail of the anterior juvenile body part from the left side view. The regular areas of low signal intensity in the images are caused by lower light intensity falling on the camera chip margins during the imaging of the tiles. a., anus; at., atrioporus; ci., buccal cirri; ch., notochord; c.n.1/2, first and second cranial nerve; g.s., gill slits; m., mouth; n.c., neural cord; sp.1/2/3, first, second, and third spinal nerves; v.n.l./v.n.r., nerve to the left/right side of the velum. Individual channels were imaged sequentially in the track switch mode—z-stack.

Next, we examined the autofluorescence of a 6-month-old specimen with 34 pairs of gill slits and length of 10.5 mm (Figure 4). The autofluorescence signals of the 3– and 6-month-old juveniles demonstrate many similarities. As in the case of younger juveniles, DAPI staining was performed to get the outline of the whole-mount specimen (Figure 4A). The green autofluorescence was the strongest in buccal cirri (Figure 4B). The signal emitted in the red spectrum showed weaker intensity and marked the notochord, buccal cirri, branchial apparatus, gut, and hepatic cecum (Figure 4C). The most obvious autofluorescence in the far-red channel was present in the hepatic cecum and gut (Figure 4D). Remarkably, optical sectioning revealed a strong signal in the notochord that could be used as endogenous marker (Figures 4F,G).

The immunostained specimen with the length of 9.5 mm came from the same batch. The immunostaining of acetylated tubulin visualized the increasing complexity of neural organization of the growing amphioxus juvenile (Figures 4H–Q and Supplementary Videos 4, 5). Imaging of cleared specimens allowed us to observe distinct cranial or spinal nerves rising from the neural cord, including branches of the second and third spinal nerves that innervate the right and left side of the velum (Figures 4M,N,P,Q). Interestingly, the innervation around the atriopore becomes profoundly reduced in the 6-month-old juvenile as compared with the 3-month-old specimen (compare Figures 3, 4). Taken together, the whole-mount IHC of cleared amphioxus larvae and juvenile specimens with anti-acetylated-α-tubulin antibodies allowed us to reveal the details of the developing amphioxus nerve net.

### Immunohistochemistry Staining of an Amphioxus 1-Month-Old Larva and a 3-Month-Old Juvenile With Anti-Melanopsin Antibody

**FIGURE 5:**
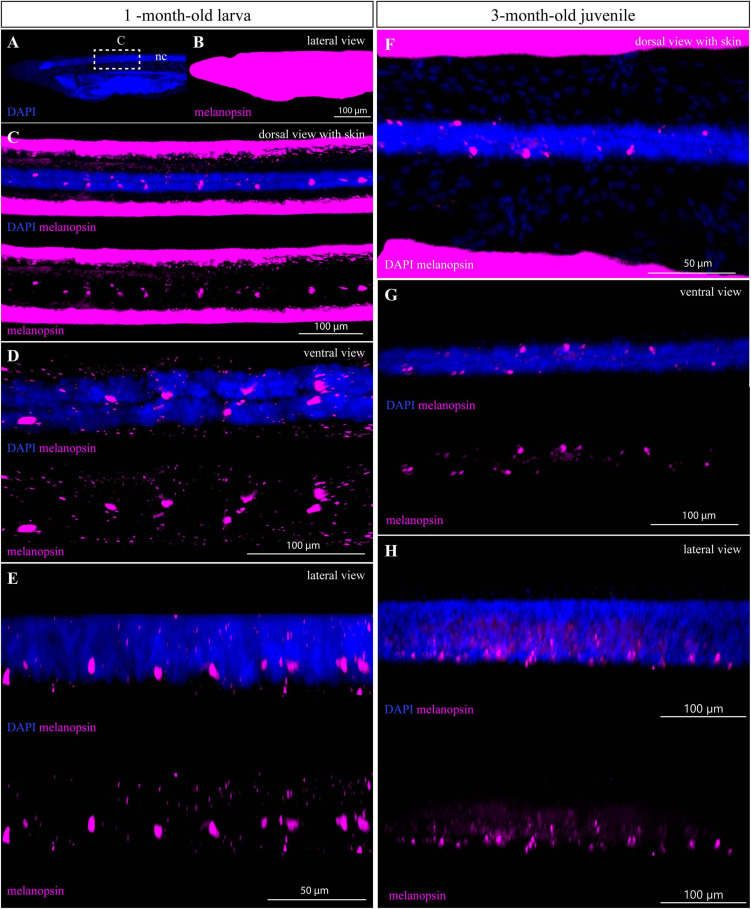
IHC staining of melanopsin in 1- and 3-month-old amphioxus specimens. **(A,B)** Whole mount of the anterior part of 1-month-old larva shown with the adjustment of brightness/contrast required to visualize the signal of melanopsin in the neural tube **(C–E)**. Dotted rectangle marks the region from which the neural tube was cropped by processing in IMARIS analysis software shown in **(C–E)**. **(C)** Subset of horizontal slices containing ventral neural cord with melanopsin-positive cells. **(D)** Subset of horizontal slices containing neural cord and melanopsin-positive cells after removal of optical slices containing skin tissue by the processing software. **(E)** Cropped area containing the neural cord with melanopsin-positive cells (with and without DAPI staining) from the lateral view. **(F)** Subset of horizontal slices with the neural tube of a 3-month-old juvenile stained with DAPI and melanopsin antibody. **(G)** Subset of horizontal slices containing the neural cord and melanopsin-positive cells after optical removal of skin tissue, from the dorsal view. **(H)** Detail of neural cord with melanopsin-positive cells (with and without DAPI staining) after removal of optical slices containing overstained skin tissue, from the lateral view. The specimens were imaged sequentially in the track switch mode—z-stack.

Next, we tested the possibility to perform whole-mount immunostaining of larger amphioxus specimens with antibodies generated against amphioxus melanopsin. As previously described, melanopsin is present in rhabdomeric photoreceptive cells (Joseph and Hesse cells) in amphioxus adults (Koyanagi et al., 2005; Pergner and Kozmik, 2017). We were able to detect the signal in Hesse photoreceptive cells in a 1-month-old larva and a 3-month-old juvenile (Figure 5). In contrast to the acetylated-α-tubulin, the intensity of melanopsin staining was weaker and required higher power of excitation laser. In addition, an undesired ectopic signal caused by non-specific sticking of the antibodies was present on the whole surface of the specimens (Figures 5A–C,F). This phenomenon was previously observed in the case of larva staining with many other antibodies (Vopalensky et al., 2012; Pergner et al., 2020). The advantage of the imaging technique based on optical sectioning lies in the possibility to observe the cells or structures hidden in the context of 3D whole mount. We visualized Hesse cells in the neural tube by removing the optical sections with skin tissue (Figures 5C–H). We were unable to detect melanopsin in the 6-month-old juveniles, most likely due to the low permeabilization of the dense tissue.

## Discussion

In this study, we demonstrated that light sheet microscopy (LSM) combined with tissue clearing enables rapid imaging of large amphioxus specimens with single-cell resolution. The main advantage of the whole-mount scanning is the possibility to examine large specimens while the tissue context is preserved. In addition, compared with confocal fluorescence microscopy, LSM imaging is faster and avoids photobleaching of deeper layers during the scanning of thick samples (Keller and Stelzer, 2008; Kromm et al., 2016).

Staining of acetylated tubulin in the developing amphioxus revealed detailed nerve branching that could be observed in the context of the whole mount and as individual sections at high resolution. Anti-acetylated tubulin antibody is frequently used by researchers to visualize the nerve net in vertebrates and invertebrates. In amphioxus, the staining was previously applied to describe the peripheral nervous system of early larvae, premetamorphic, metamorphosing, and advanced postmetamorphosis individuals (Kaji et al., 2001; Yasui and Kaji, 2008; Kaji et al., 2009; Yasui et al., 2014; Kaji et al., 2016) or neural organization of the brain in the adult (Castro et al., 2015). These earlier studies performed confocal imaging of selected segments or individual paraffin sections. The cytoarchitecture of the central nervous system of late premetamorphic larva, postmetamorphic juvenile, and adult amphioxus was studied with classical histological methods and by immunohistochemistry using acetylated tubulin (Lacalli et al., 1994; Lacalli, 2000, 2003; Ekhart et al., 2003; Castro et al., 2015). In 3– and 6-month-old juveniles, we identified several cell types that have been described previously, including anterolateral migrated cells, transluminal cells, somatic motoneurons, lamellate cells, and Retzius bipolar cells (Supplementary Figure 1).

Compared with acetylated tubulin, the whole-mount staining of melanopsin was weaker, and we were unable to detect any signal in 6-month-old juveniles. Generally, detection of the signal in internal structures within whole-mount staining of older specimens is always challenging due to the high density of the sample. Prolonging the incubation time with antibody together with permeabilization methods may help to overcome this obstacle. In amphioxus, melanopsin is expressed in photoreceptor cells of the Hesse organ (also called dorsal ocelli) and Joseph cells (Koyanagi et al., 2005; Pergner and Kozmik, 2017). While the first dorsal ocelli are already developing in the ventral part of the neural tube at the mid-neurula stage, Joseph cells have so far been identified in the dorso-caudal region of the cerebral vesicle only in the adult amphioxus (Ruiz and Anadon, 1991; Lacalli et al., 1994; Koyanagi et al., 2005; Castro et al., 2015). In 3-month-old juveniles, we detected melanopsin only in the dorsal ocelli, which is consistent with the idea that Joseph cells develop later during amphioxus ontogenesis.

Autofluorescence often complicates the analysis of fluorescently stained specimens. The amphioxus genome encodes a variety of GFP-like proteins with distinct spectral properties (Deheyn et al., 2007; Bomati et al., 2009; Yue et al., 2016). Although the CUBIC clearing procedure preserves the signal of endogenous fluorophores, the intensity of autofluorescence may be affected by any chemicals during fixation and staining. In this study, we observed a weak signal of endogenous fluorescence across the spectrum with the strongest one in the green channel. The high variability in the autofluorescence patterns among the different developmental stages is intriguing. Bomati et al. (2009) identified 16 GFP-like genes in the amphioxus genome belonging to six clades. Individual GFPs not only possess different spectral characteristics but their expression is also highly dynamic during development. Besides, it was shown that GFPa1 is selectively expressed in the oral cirri of the adults suggesting that some GFPs may also display the tissue-specific expression (Deheyn et al., 2007). The absorbance spectrum among different GFPs ranges from 375 to about 525 nm with a peak between 470 and 504 nm. The weak autofluorescence in the blue spectrum may be explained by the ability of some GFPs to be excited at 405 nm. Detailed description of the scanning parameters and characterization of the autofluorescence of distinct structures may help researchers to choose the secondary antibody with a suitable fluorophore. At the same time, endogenous fluorescence can be used as counterstaining or as a marker to observe the cells or structures of a specimen without additional staining. In fact, the autofluorescence of individual structures may be used not only to study the intact animals but also to analyze the phenotypes after gene perturbations or chemical treatment.

For example, the notochord of 3– and 6-month-old juveniles is nicely visualized by setting the excitation wavelength at 638 nm. The parameters of notochord autofluorescence correlate with the spectral properties of porphyrins. Porphyrins are present in different tissues and exhibit a broad absorption spectra with one peak around 400 nm and four peaks between 490 and 650 nm. The emission spectra of porphyrins are located between 550 and 700 nm (Plus, 1992). Porphyrins constitute a part of hemoglobin protein subunits. The presence of hemoglobin in the notochord of amphioxus was demonstrated by high-performance liquid chromatography and by measuring optical absorption spectra (Bishop et al., 1998). The range of absorption spectra of the notochord sample was from 400 to 650 nm. We therefore speculate that the observed notochord autofluorescence may be caused by the presence of hemoglobin or some other proteins associated with porphyrins.

An important issue in SPIM imaging is the production of large amounts of data and, thus, the need for high-performance computational technology and large data storage capabilities. In addition, higher amounts of data require more time for their processing. Therefore, careful planning of the experiment in terms of the used resolution, zoom, volume, and number of channels should be performed before the scanning itself (Amat et al., 2015; Kromm et al., 2016). For example, the whole mount of the specimen can be imaged at a lower zoom level and the details with higher magnification (Supplementary Table 1).

Taken together, in this study, we demonstrated that LSM may be applied to study the whole mount of large amphioxus specimens at high resolution. We believe that this technique will constitute a valuable complement to the conventional methods of electron (Lacalli et al., 1994, 1999; Wicht and Lacalli, 2005) and confocal microscopy (Yasui and Kaji, 2008). Moreover, the possibility to visualize the entire amphioxus body at the single-cell resolution may facilitate unbiased identification of the morphological and molecular differences between wild-type and experimentally manipulated animals.

## Data Availability Statement

The raw data supporting the conclusions of this article will be made available by the authors, without undue reservation.

## Author Contributions

SM, HC, ZK, and IK designed the study and conceived the experiments. SM, HC, and AV performed the wet lab experiments. SM, HC, AV, and IK analyzed the data. IK and ZK provided new reagents and animal specimens. SM and IK wrote the manuscript. All authors have read and approved the manuscript.

## Conflict of Interest

The authors declare that the research was conducted in the absence of any commercial or financial relationships that could be construed as a potential conflict of interest.

## Publisher’s Note

All claims expressed in this article are solely those of the authors and do not necessarily represent those of their affiliated organizations, or those of the publisher, the editors and the reviewers. Any product that may be evaluated in this article, or claim that may be made by its manufacturer, is not guaranteed or endorsed by the publisher.

## Supplementary Material

Supplementary Figure 1 Selected optical sections show different types of neurons immunostained with anti-acetylated tubulin in 3-month-old juvenile **(A-C”)** and 6-month-old juvenile **(D-E”)**. **(A-A”)** Anteriolateral migrated cells. **(B-B”)** Translumenal cells. **(C-C”)** Somatic motoneurons. **(D-D”)** Lamellate cells. **(E-E”)** Retzius bipolar cells. Arrows in the individual panel point to indicated cell type. Faint vertical and horizontal lines indicate the plane of section.

Supplementary Table 1 Technical parameters of the imaging of amphioxus specimens.

Supplementary Table 2 Technical specification of analysis computers.

Supplementary Video 1 X-/y-axis rotation of 3D reconstruction of 1-month-old larva immunostained with anti-acetylated tubulin (green) antibody with DAPI (blue).

Supplementary Video 2 Removal of optical horizontal slices out of 1-month-old larva immunostained with anti-acetylated tubulin (green) antibody with DAPI (blue).

Supplementary Video 3 Rotation of 3D whole mount and close-ups of the parts of 3-month-old larva immunostained with anti-acetylated tubulin (green) antibody with DAPI (blue). (0:00:00–0:00:10 s) X-/y-axis rotation of the 3D whole mount. (0:00:11–0:00:20 s) buccal cirri highlighted by the adjustment of the brightness/contrast. (0:00:20–0:00:50 s) slow-motion focusing on details of the nerve net from the lateral view.

Supplementary Video 4 Immunostaining of 6-month-old juvenile I with anti-acetylated tubulin antibody (green) and DAPI (blue). (0:00:00–0:00:36 s) 3D whole mount and slow-motion of the parts of the specimen from the lateral view. (0:00:37–0:00 s) Slow-motion of 3D whole mount from the ventral view.

Supplementary Video 5 Immunostaining of 6month-old juvenile II with anti-acetylated tubulin antibody (green) and DAPI (blue). (0:00:00–0:00:05 s) Rotation of 3D whole mount to the ventral view (0:00:06–0:00:18 s) Slow-motion of 3D whole mount from the ventral view (0:00:19–0:00:36 s) Slow-motion of the specimen from the dorsal view gradually rotated to the lateral view after removal of ventral slices by image processing. (0:00:37–0:00:50 s) X-/z-rotation of 3D whole mount from the lateral to frontal view.
