# Supplementary figures and images for: Optical Clearing and Light Sheet Microscopy Imaging of Amphioxus
Source: Front Cell Dev Biol. 2021 Jul 26;9:702986. doi: 10.3389/fcell.2021.702986 (PMC8350520; doi:10.3389/fcell.2021.702986)

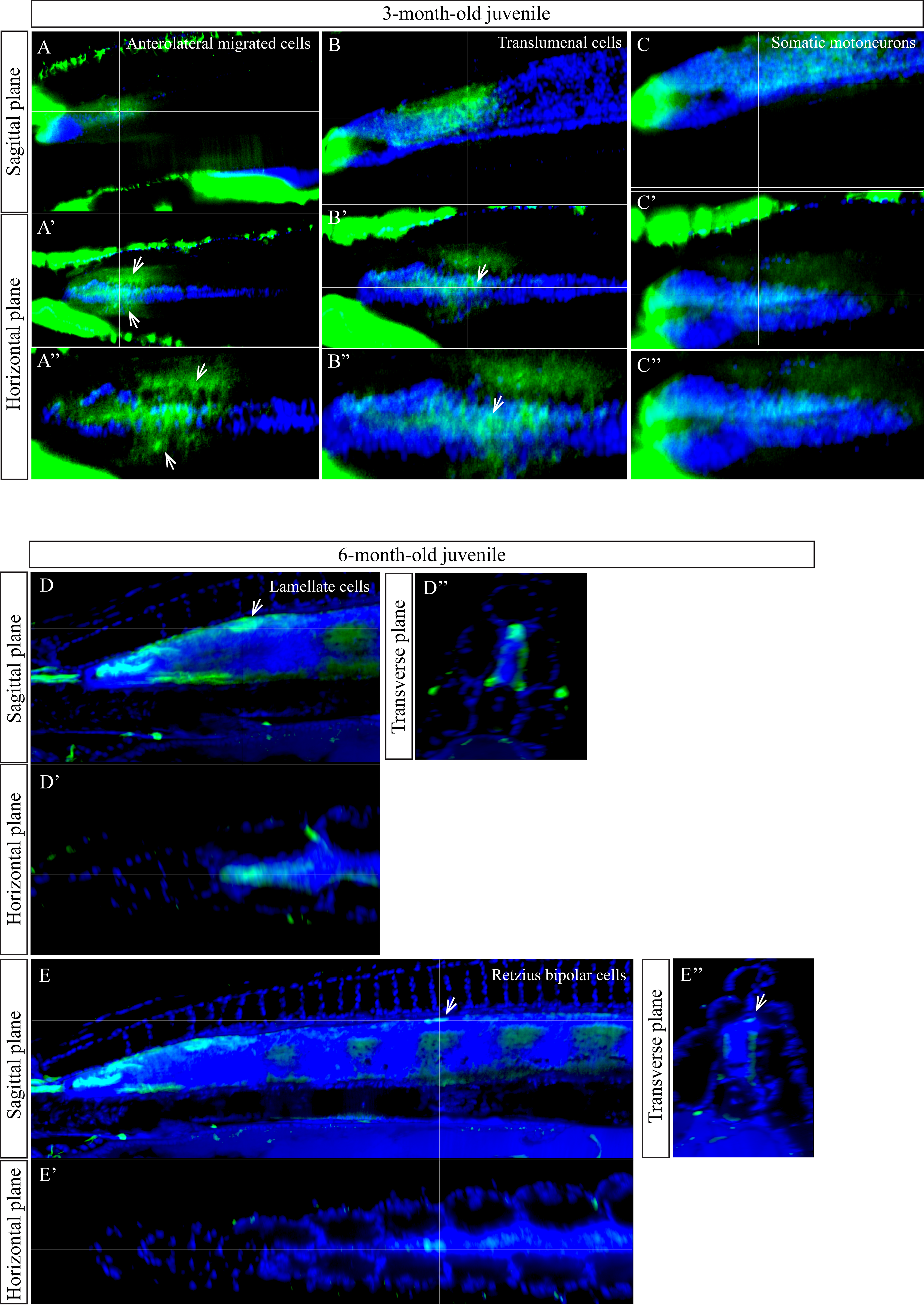

Supplement: Supplementary Figure 1 — Selected optical sections show different types of neurons immunostained with anti-acetylated tubulin in 3-month-old juvenile (A-C”) and 6-month-old juvenile (D-E”). (A-A”) Anteriolateral migrated cells. (B-B”) Translumenal cells. (C-C”) Somatic motoneurons. (D-D”) Lamellate cells. (E-E”) Retzius bipolar cells. Arrows in the individual panel point to indicated cell type. Faint vertical and horizontal lines indicate the plane of section. [file Image_1.TIF]
