# Supplementary material for: Optical Clearing and Light Sheet Microscopy Imaging of Amphioxus
Source: Front Cell Dev Biol. 2021 Jul 26;9:702986. doi: 10.3389/fcell.2021.702986 (PMC8350520; doi:10.3389/fcell.2021.702986)
Supplement: Supplementary Table 1 — Technical parameters of the imaging of amphioxus specimens. [file Data_Sheet_1.PDF]

	autofluorescence			immunohistochemistry				
	1-m-old	3-m-old	6-m-old	1-m-old (1)	1-m-old (2)	3-m-old	3-m-old	6-m-old
animal length (mm)	2.9	8	10.5	2.7	3.5	8	detail	9.7
Channels	B,G,R,M	B,G,R,M	B,G,R,M	B,G,M	B,G,M	B, G	B,G,M	B, G
Zoom	0.8	0.4	0.4	0.8	0.8	0.8	0.8	0.4
Image dimensions	1920 x 1920	1920 x 1920	1920 x 1920	1920 x 1920	1920 x 1920	1920 x 1920	1920 x 1920	1920 x 1920
Image size (mm)	1.48 x 2.44	2.95 x 8.65	5.8 x 10.55	1.48 x 2.44	2.44 x 2.44	1.95 x 8.12	0.53 x 0.53	2.95 x 10.55
Pixel size (µm)	0.27 x 0.27	0.55 x 0.55	0.55 x 0.55	0.27 x 0.27	0.27 x 0.27	0.27 x 0.27	0.27 x 0.27	0.55 x 0.55
Z-step (µm)	0.9	2	2	0.9	0.9	0.9	0.9	2
Z thickness (µm)	935	1566	2800	934	515	958	202	2852
Tiles	15	27	35	15	25	68	-	29
Total time (min)	121	156	381	132	112	251	3.5	321
Final file size (GB)	**184.20**	**254.82**	**535.66**	**124.41**	**134.52**	**460.58**	**3.07**	**234.20**
