# Supplementary material for: Optical Clearing and Light Sheet Microscopy Imaging of Amphioxus
Source: Front Cell Dev Biol. 2021 Jul 26;9:702986. doi: 10.3389/fcell.2021.702986 (PMC8350520; doi:10.3389/fcell.2021.702986)
Supplement: Supplementary Table 2 — Technical specification of analysis computers. [file Data_Sheet_2.pdf]

Supplementary table 2: PC for analysis of the light sheet data
(important values are highlighted)

Type I	Windows 10 Education (2004), 64-bit; **PROCESSOR** 1x8 core i7-7820X, 3.6 GHz (**Pasmark 18920, CPU-Z 4649**); **RAM 128GB** DDR4 2133 MHz; GeForce GTX 1080 Ti 11 GB; SYSTEM - 1TB Nvme M.2 SSD, Read 1.22 GB/s, Write 1.29 GB/s; CACHE - 1TB Nvme M.2 SSD, Read 1.59 GB/s, Write 1.7 GB/s; **DATA - 4 TB** SAS RAID0, 4x SATAIII SSD, Read 1.51 GB/s, Write 1.35 GB/s; 10 Gb/s Ethernet
Type II	Windows 10 Education (20H2), **PROCESSOR** 2x12 core e5-2620, 2.4 GHz (**Pasmark 16139, CPU-Z 4246**); **RAM 128GB** DDR4 2133 MHz; 1x Nvidia Quadro K2200 4GB; SYSTEM - 256 MB SSD, Read 0.42 GB/s, Write 0.40 GB/s; PAGEFILE - 256 MB SSD, Read 0.42 GB/s, Write 0.39 GB/s; CACHE - 1TB Nvme M.2 SSD (warranty 5 Years or 1,200 TBW), Read 1.765 GB/s, Write 1.679 GB/s; **DATA - 32 TB** SAS RAID5, 8x SATAIII HDD, Read 1.14 GB/s, Write 1.25 GB/s; 2x10 Gb/s ethernet
